# Structural Studies Providing Insights into Production and Conformational Behavior of Amyloid-β Peptide Associated with Alzheimer’s Disease Development

**DOI:** 10.3390/molecules26102897

**Published:** 2021-05-13

**Authors:** Anatoly S. Urban, Konstantin V. Pavlov, Anna V. Kamynina, Ivan S. Okhrimenko, Alexander S. Arseniev, Eduard V. Bocharov

**Affiliations:** 1 Shemyakin–Ovchinnikov Institute of Bioorganic Chemistry RAS, 117997 Moscow, Russia; anatoly.urban@gmail.com (A.S.U.); aneskaminina@mail.ru (A.V.K.); 2 Federal Clinical Center of Physical-Chemical Medicine of FMBA, 119435 Moscow, Russia; qpavlov@mail.ru; 3 Research Center for Molecular Mechanisms of Aging and Age-related Diseases, Moscow Institute of Physics and Technology, 141701 Dolgoprudny, Russia; i.s.okhrimenko@gmail.com

**Keywords:** Alzheimer’s disease, amyloid precursor protein, amyloid-β peptide, structural–dynamical properties, toxic oligomerization, molecular mechanism, neuronal membrane, protein–protein and protein–lipid interactions, bioactive peptides for therapy and diagnosis

## Abstract

Alzheimer’s disease is the most common type of neurodegenerative disease in the world. Genetic evidence strongly suggests that aberrant generation, aggregation, and/or clearance of neurotoxic amyloid-β peptides (*Aβ*) triggers the disease. *Aβ* accumulates at the points of contact of neurons in ordered cords and fibrils, forming the so-called senile plaques. *Aβ* isoforms of different lengths are found in healthy human brains regardless of age and appear to play a role in signaling pathways in the brain and to have neuroprotective properties at low concentrations. In recent years, different substances have been developed targeting *Aβ* production, aggregation, interaction with other molecules, and clearance, including peptide-based drugs. *Aβ* is a product of sequential cleavage of the membrane glycoprotein APP (amyloid precursor protein) by β- and γ-secretases. A number of familial mutations causing an early onset of the disease have been identified in the APP, especially in its transmembrane domain. The mutations are reported to influence the production, oligomerization, and conformational behavior of *Aβ* peptides. This review highlights the results of structural studies of the main proteins involved in Alzheimer’s disease pathogenesis and the molecular mechanisms by which perspective therapeutic substances can affect *Aβ* production and nucleation.

## 1. Introduction

Alzheimer’s disease (AD) predominantly affects the elderly and progressively worsens with age, currently accounting for 60–70% of cases of dementia worldwide. With the ongoing increase in life expectancy, the number of people aged over 65 is predicted to triple by 2050, making this debilitating ailment not only a serious challenge to the healthcare system but also a complex social and economic issue [1]. Clinical manifestations of AD are attributed to selective degeneration of neurons in the regions of the cerebral cortex responsible for cognitive perception and memory. The origin, pathogenesis, and treatment options have been in the focus of multidisciplinary studies over the last decades; however, the pharmaceutical treatment strategies available to date have only a limited effect in slowing down the disease progression rather than restoring the impaired cognitive functions. Based on the clinical evidence, the disease symptoms are associated with the formation of aggregates of two proteins: extraneuronal senile plaques and intraneuronal neurofibrillary tangles (NFTs) [2,3] consisting of oligomerized amyloid-β peptides (*Aβ*) and phosphorylated tau-proteins, respectively. Besides that, multiple correlations of AD initiation and/or development with various lipid metabolism abnormalities have been found [4,5].

Three major hypotheses were historically proposed in relation to causal factors of AD development [6]. The earliest of the three was the so-called cholinergic hypothesis associating the disease with impaired synthesis of acetylcholine neuromediator. However, based on clinical studies, all the treatment strategies relying on this hypothesis proved to be mostly symptomatic and incapable of curing the disease to complete recovery of patients [7]. Another hypothesis associated the disease development with aberrations in the folding of tau proteins, whose normal function is to stabilize tubulin microtubules playing diverse roles in regulation of neuron morphology through cytoskeleton formation and intraneuronal cargo trafficking. Tau protein affinity to microtubules is regulated by its phosphorylation, and hyperphosphorylation causes the protein to aggregate, forming large fibrils and neurofibrillary tangles destabilizing the system of microtubules, impairing intracellular trafficking, and ultimately causing neuron death [8]. Nevertheless, the more recent genetic evidence strongly suggests that the disease is triggered by abnormal generation and/or clearance of the neurotoxic *Aβ* produced via sequential extramembrane and intramembrane cleavage of a transmembrane (TM) amyloid precursor protein (APP) by α-, β-, and γ-secretases [3,9]. Accordingly, the “amyloid hypothesis” attributing the main cause of the disease to aberrant production, clearance, and/or conformational distribution of *Aβ* and especially its oligomeric forms [10,11] has become predominant. The supporting genetic evidence includes the fact that the familial mutations associated with the early onset of AD are mostly localized in the genes encoding APP or the secretases involved in its proteolysis [3,12,13]. Circumstantial supporting evidence for this hypothesis comes from the studies of patients afflicted with Down syndrome who have an extra copy of chromosome 21 [14] and, among other abnormalities, produce 1.5 times as much APP as other people. By the age of 40, almost 100% of people with Down syndrome who die have the changes in the brain commonly associated with AD. A significant role of apolipoprotein E (ApoE) in the disease progression is also consistent with the amyloid hypothesis. Indeed, the presence of the E4 allele of the ApoE gene correlated with amyloid overexpression and accumulation is known to be among the most significant contributors to AD development, simultaneously increasing its risk and causing earlier onset, along with rare familial mutations [2,4]. Although several reasons may underlie the effect of specific apoE isoforms on AD pathogenesis, convincing evidence suggests that the physical interaction of apoE with *Aβ* plays an important role. APP and its derivatives, including *Aβ* isoforms, soluble ectodomain, and C-terminal fragments, definitely play diverse, although not fully known and completely understood, roles in neuronal tissue function and homeostasis. Different *Aβ* isoforms normally occur in the healthy human brain regardless of age and are apparently essential for brain function, participating in synaptic signal transduction, neuroplasticity, and inflammatory response [15,16,17,18,19].

This review highlights the results of recent structural studies of the major currently known factors of AD pathogenesis, as well as the molecular mechanisms by which perspective therapeutic substances affect *Aβ* production and oligomerization.

## 2. Diverse Biological Function of the Amyloid Precursor Protein

Amyloid precursor protein (APP), expressed in the cells of virtually all animal tissues and having a pronounced evolutionary conservatism, shares structural and functional similarities with type I membrane-embedded receptors [20]. It was hypothesized to act as a cholesterol receptor in neuronal membrane rafts [21] and to participate in intracellular signaling [20], regulation of neuronal iron metabolism [22], and inflammatory processes [17]. APP gene consists of 20 exons, and its alternative splicing results in the generation of over 10 different isoforms in different tissues, APP695 being predominant in the neuronal tissue. Pronounced evolutionary conservatism of the APP genes implies that the corresponding proteins play vitally important biological functions. Indeed, knockout of *APL-1* (APP homolog) gene in *C. elegans*, commonly used as a model organism, results in a lethal phenotype [23]. Expression of a truncated *APL-1* form without the cytoplasmic domain, however, is sufficient for survival [24]. In *D. melanogaster*, knockout of *APPL* (APP-like protein) gene is not lethal but causes aberrant synapse morphology and abnormal behavioral patterns [25].

In vertebrates, the APP gene family includes two APP homologs besides the APP itself, *APLP1* and *APLP2*, with partly overlapping functions and a variety of biologically active products resulting from complex proteolytic processing. For example, *APP* knockout mice proved to be viable and fertile, though having multiple abnormalities including reduced body weight [26]; elevated concentrations of copper [27], cholesterol, and sphingolipids [28] in brain tissues; and multiple neurological impairments. These impairments included decreased size of the frontal lobe commissures and corpus callosum agenesis, corroborating the hypothesis of APP involvement in neurite outgrowth and axonal pathway formation [29]. Interestingly, in this case, all the abnormalities are abolished by the expression of an abbreviated soluble form of APP, sAPPα, corresponding to a product of APP proteolysis by α-secretase [30], implying that this processing pathway is essential for the normal development and function of the neural system. Experiments with knockout of individual APP homologs or their combinations proved partial functional overlap between the homologs and their importance for the formation of intercellular contacts and interactions, as well as neurotrophic and neuroprotective functions [31,32]. The role of APP in intercellular interactions is further corroborated by structural studies. In particular, APP ectodomains of the same cell or two neighboring cells were shown to be capable of forming heparin-mediated *cis-* and *trans-*dimers [33]. Besides, APP shares a common proteolytic cleavage mechanism with type I receptors of the Notch family [34], which supports the hypothesis of APP involvement in interactions with neighboring cells and extracellular factors. Likewise, certain interactions of APP with intracellular and plasma membrane components also have functional implications. For example, its ectodomain interacts with membrane-associated prion protein PrP [20,35], whereas its C-terminal segment participates in the cytoplasmic signaling cascades. APP was shown to directly interact with cholesterol molecules in cellular membranes [34,36,37], with multiple lines of evidence implying that such interactions are biologically relevant. These include clinical evidence of cholesterol metabolism abnormalities in AD patients, results of biophysical experiments indicative of competition between cholesterol binding and APP dimerization, and preferential localization of APP and γ-secretases in cholesterol-enriched lipid rafts [38,39,40].

## 3. Multidomain Structure of the Amyloid Precursor Protein

**Figure 1:**
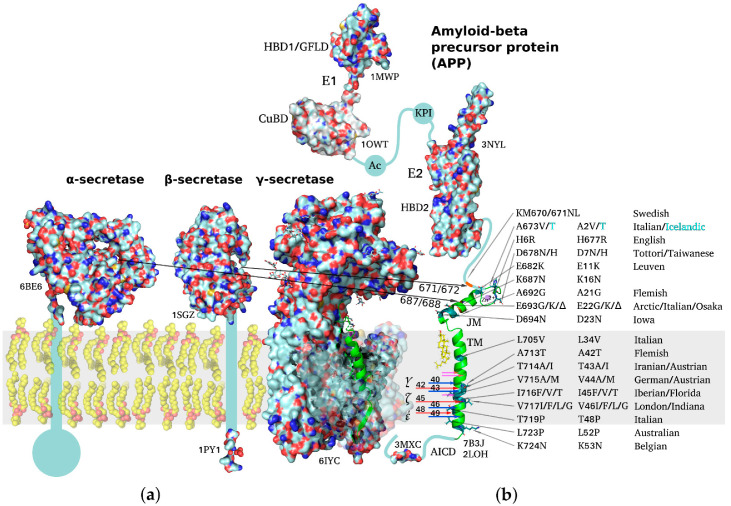
Schematic representation of structures of amyloid precursor protein and α-, β-, and γ-secretases responsible for *Aβ* production. PDB accession number is indicated for each molecule throughout the figure. (**a**) Molecular surfaces of the structural elements of α-, β-, and γ-secretases. Single-span transmembrane (TM) domains of α- and β-secretases are shown as bars where high-resolution structure is unavailable. (**b**) Full-length APP structure based on individually resolved structures of its parts: flexible intracellular C-terminal domain (AICD), TM domain, connected through a flexible extracellular juxtamembrane (JM) region (containing *Aβ* metal-binding domain) to an ectodomain consisting of (i) E1 subunit including a cysteine-rich growth factor-like domain (HBD1/GFLD) and a copper/zinc-binding domain (CuBD), an acidic region (Ac), a Kunitz-type protease inhibitor domain (KPI), and (ii) E2 subunit with a second heparin-binding domain (HBD2). Resolved domain structures are shown as ribbon diagrams, and unstructured flexible connecting loops are shown as solid lines. The familial mutations attributed to increased risk or earlier age of AD development are shown in black on the TM and JM segments. A673T mutation decreasing APP proteolysis by β-secretases is highlighted in cyan. Sites of cleavage by α-, β-, and γ-secretases are indicated by arrows color-coded to distinguish between two alternative cleavage cascades generating *Aβ*_1–42_ and *Aβ*_1–40_ peptides (48 > 45 > 42 vs. 49 > 46 > 43 > 40). Cholesterol molecule interacting with the N-terminal part of APP TM helix is shown. The inset demonstrating the helical APP TM domain (in green) with a C-terminal turn (3 a.a. residues) unfolded into a β-strand is shown in the γ-secretase active center.

APP is a classical type I multidomain protein with a single TM span, consisting of intracellular, extracellular, and TM parts, capable of lateral dimerization in the plasma membrane and adopting several alternative functional conformations. The following structural domains can be distinguished in unprocessed APP molecules: E1 subunit composed of a heparin-binding domain along with a cysteine-rich growth factor-like domain (HBD1/GFLD) and a copper/zinc-binding domain (CuBD), an acidic region (Ac), a Kunitz-type protease inhibitor domain (KPI, not present in APP695), E2 subunit with a second heparin-binding domain (HBD2), a random coil juxtamembrane (JM) region and TM domain (including the *Aβ* sequence), and an intracellular C-terminal domain (AICD) (Figure 1) [20,41,42]. APP dimerization is known to be induced by the N-terminal E1 region, the connecting loop between the HBD1/GFLD and CuBD formed by disulfide bridges being essential for stabilization of the homodimeric state, while JM and TM regions also participate in homodimerization [20,43,44]. Due to the relatively large size (~750 a.a. residues) of the protein, its ability to dimerize in a membrane environment, and domain mobility with respect to each other, no high-resolution structures of the full-size APP have been obtained to date, spatial structures of the domains having been resolved separately via different structural methods.

Besides performing apparently essential biological functions in its unprocessed form, APP also serves as a predecessor for multiple biologically active products generated through different cleavage pathways. For example, cleavage by α-secretase yields soluble sAPPα, an important neurotrophic factor enhancing proliferation of neuronal stem cells and modulating synaptic plasticity [45,46,47]. A similar β-secretase cleavage product sAPPβ devoid of the *Aβ* metal-binding domain has no neuroprotective functions of the sAPPα, but was shown to interact with DR6 receptor (“death receptor 6” of the TNFα family), inducing caspase 6 activation and axon pruning. Although no high-resolution structure of the entire ectodomain is available so far, sAPPα structure was resolved by small-angle X-ray scattering [48,49,50] in combination with molecular modeling, fitting the known spatial structures of GFLD, CuBD, KPI, and E2 domains into the low-resolution structure of the APP ectodomain [48,51].

APP TM domain contains the sequences of the β-amyloid peptides, excessive accumulation of which is correlated with advanced AD stages. Moreover, most of the familial mutations associated with early onset of the disease are situated within the TM domain or the immediately adjacent JM regions. The TM domain proper includes residues 700–723 forming a hydrophobic span; however, it is often binned together with the JM region 686–699, which also participates in interactions with the membrane, and with 672–685 metal-binding domain [52,53]. This membrane-associated domain was extensively investigated by NMR spectroscopy methods, including high-resolution and solid-state techniques [54,55,56]. Labile conformation of the metal-binding domain was shown to be modulated by intra- and interprotein Zn^2+^ binding. The JM region can form a flexible amphiphilic helix submerging under the membrane surface or a β-strand depending on the environment, and this transition can underlie nucleation of amyloid aggregates. A flexible loop connects the JM region to the TM domain, which includes a kink region (following Gly708/Gly709 repeat) responsible for the sensitivity of the TM domain conformation to the membrane environment or point mutations [21,54,56,57,58,59,60]. High-resolution dimeric structure of the α-helical APP TM segment corresponding to *Aβ_15–55_* was first obtained in [43] in DPC micelles used as a membrane environment model. However, the possibility of alternative dimerization of the TM domain was suggested by a number of studies, e.g., in lipid bilayers [61,62]. The presence of two or more dimerization motifs in the TM segment is common for type I receptors, often enabling transition between different functional states of the receptor, e.g., between the dormant and ligand-activated states. Evidence is available linking APP TM and/or extracellular domain dimerization with the choice of proteolytic cleavage pathway [63]. The familial mutations associated with early AD onset localized within the TM or JM region were shown to be capable of altering APP recognition and cleavage by secretases and/or affecting oligomerization and toxicity of mature *Aβ* species at the expense of global or local conformational rearrangements and intermolecular interactions of varying degree of specificity [58,60,64,65,66,67,68].

Like many intracellular domains of diverse TM proteins, the intracellular AICD domain composed of about 50 residues does not have a stable ordered structure, as demonstrated by solution NMR spectroscopy [69]. However, as is the case with many intrinsically disordered proteins (IDPs), the measured backbone NMR chemical shifts are indicative of transient secondary structure elements, which can serve as precursors of the structure assumed by the domain upon interaction with intracellular ligands or certain components of the cytoplasmic membrane leaflet [70,71]. Overall, AICD is responsible for the intracellular stage of signal transduction and APP trafficking towards the plasma membrane. For example, after being cleaved by γ-secretase, AICD in complex with Fe65 protein is transported into the nucleus where it regulates expression of several microRNA species, with AICD overexpression inhibiting differentiation of stem cells into neurons, mostly through increased expression of mir-663 [72]. More recently, several G-protein-dependent pathways were also hypothesized to be relevant to APP biological function [73].

## 4. Proteolytical Processing of the Amyloid Precursor Protein

The canonic APP proteolytic processing pathways consist of sequential extramembrane and intramembrane cleavage first by α- or β-secretases and then by γ-secretases (Figure 1), yielding diverse *Aβ* isoforms depending on the exact cleavage pattern [3,20,74]. Initially, APP can be cleaved by a membrane-bound β-secretase, aspartyl protease BACE (β-site APP-cleaving enzyme), removing a large APP ectodomain and yielding a membrane-bound 99 amino acid C-terminal fragment (CTF). Thereafter, this C99 or APP CTFβ fragment undergoes further sequential cleavage by a large membrane protein complex known as γ-secretase. This intramembrane aspartyl protease complex starts processing C99 in the TM domain at the ε-site, thereby releasing the APP intracellular domain (AICD) from the membrane into the cytosol, initiating an intracellular signaling cascade. A spectrum of *Aβ* isoforms is generated by downstream γ-secretase cleavages at the ζ- and γ-sites, truncating the APP TM domain until sufficiently short, predominantly 38 to 42 residues long, and *Aβ* isoforms are released from the membrane into the extraneuronal space or into the lumen of secretory pathway organelles. Normally the major ultimate cleavage product is *Aβ*_1–40_, the shorter *Aβ*_1–38_ and the longer *Aβ*_1–42_ being present as minor components. Onset of AD, however, is associated with an overall increase in production of all *Aβ* isoforms and a shift of distribution between the isoforms towards a higher fraction of *Aβ*_1–42_ that is more hydrophobic and fibrillogenic than *Aβ*_1–40_. All *Aβ* isoforms are capable of associating into cytotoxic oligomers interacting with neuronal membranes and membrane receptors; further aggregation results in the formation of fibrils depositing as the senile plaques observed at the late stages of AD progression. Generation of *Aβ* is competitively inhibited by an alternative extramembrane cleavage of APP mediated by membrane-bound α-secretase, a metalloprotease of the ADAM (A Disintegrin And Metalloproteinase) family, which removes the N-terminal metal-binding region of *Aβ* and generates a shorter APP CTFα, C83, further processed by γ-secretase into non-pathogenic P3 peptides.

Competition between α- and β-secretase processing of APP at the initial stage defines the subsequent cleavage cascade and spectrum of products, which suggests a potential AD treatment strategy based on β-secretase inhibition and/or α-secretase upregulation [75,76]. The α-secretase belongs to ADAM metalloproteases, type I transmembrane proteins having modular domain structure. The substrates of extracellular ADAM metalloproteases include growth factors, cytokines, chemokines, receptors, and adhesion factors; thus, the metalloproteases play important roles in intracellular adhesion and signal transduction [77]. ADAM9 and ADAM17 species can process APP; however, the dominant pathway leading to p3 and sAPPα production involves ADAM10 [78,79], the substrates of which, besides APP, include Notch receptor, Eph receptors, HER receptors, TNF-α, and other membrane proteins. Several mutations associated with late onset of AD were found to be localized in ADAM10 prodomain facilitating proper folding of its ectodomain [80]. The mechanisms underlying the metalloprotease specificity are being intensively investigated, the structure of the full-size ectodomain of human ADAM10 having been recently resolved with the aid of X-ray diffraction technique [75].

In neuronal tissues, APP cleavage in the β-site is predominantly performed by BACE1 species of β-secretase, its activity increasing with age or in case of AD development [81]. Its close homolog BACE 2 was also recently implicated in AD pathogenesis [82]. Preferentially expressed in neurons, BACE1 is known to be necessary for normal axon myelinization [83], presumably in relation to its activity towards Neuregulin 1 (Nrg1) substrate [84]. Other BACE1 substrates performing essential biological functions include Notch Jag1 [85]; Navβ2 regulatory subunit of voltage-gated sodium channels [86]; KCNE1 and KCNE2, regulating voltage-gated potassium channels [87]; and Sez6L protein, associated with seizure activity [76]. BACE-1 is a type 1 integral membrane glycoprotein with a bulky ectodomain, a single TM span, and a small intracellular fragment [88]. Interaction of BACE1 with lipid bilayer is critically important for APP processing, BACE1 with deleted TM segment being inactive towards APP in vivo, BACE1 structure provides important clues for understanding the pathogenicity of the “Swedish” familial mutation APP KM670/671NL occurring in the immediate vicinity of the cleavage β-site and causing enhanced interaction of the substrate with β-secretase [45]. Another APP mutation, A673T in the immediate vicinity of the cleavage β-site, was also described, decreasing APP proteolysis by β-secretases and the likelihood of spontaneous AD development [89]. BACE1 TM domain is also distinguished by a unique trimerization motif capable of interacting with copper ions [46]. The correlation between enhanced BACE1 activity and AD development prompted a number of attempts to find safe clinically effective inhibitors of the enzyme. Over 400 spatial structures of BACE1 complexes with diverse inhibitors have been obtained, some of them currently undergoing clinical trials.

Initial APP cleavage by α- or β-secretases enables subsequent γ-secretase processing, removing the steric constraints and providing access to the γ-secretase cleavage site in the membrane interior [90,91]. Having an apparently limited specificity, γ-secretase is known to process dozens of proteins [92], including Notch receptors [34] and APP. The low specificity of the γ-secretase complex is corroborated by a small degree of homology between different substrates and the presence of amino acid residues with diverse physicochemical properties in their cleavage sites. The γ-secretase processing frequently occurs in the cholesterol-enriched endosomal membranes at low pH values [5,21,88,93,94].

The γ-secretase complex is a multidomain enzyme composed of four transmembrane proteins: presenilin (PS, in the form of PS1 or PS2 protein), nicastrin (NCT), anterior pharynx-defective 1 (Aph1), and presenilin enhancer 2 (PEN2) [95]. The diversity of γ-secretase substrates and their involvement in a broad range of cellular processes, along with the fact that the majority of familial mutations associated with AD development affect PSEN1 and PSEN2 subunits [96], stimulate their extensive investigation. The mechanisms of APP mutations affecting ε-, ζ-, and γ- cleavage sites are also apparently based on induced changes in the γ-secretase sequential cleavage process. Sequential removal by γ-secretase of 3–4 a.a. residue-long segments (one turn of a TM domain helix) from the substrate C-terminal side results in the generation of a pool of APP processing products of different lengths [74,97] (Figure 1). The exact mechanism of substrate recognition by γ-secretase has not been fully described, but it appears to involve considerable conformational rearrangements of both the enzyme and the substrate [98]. In order to enable cleavage by a γ-secretase, the C-terminal helical parts of *Aβ* precursors (APP TM domain fragments) need to unfold into extended conformation and submerge into the membrane to the depth corresponding to the location of γ-secretase active center situated near the cytoplasmic leaflet within accessibility of water molecules. Accordingly, changes in the local membrane environment or mutations affecting the accessibility of C-termini of *Aβ* precursors to the active center can alter the cleavage rate and the likelihoods of initiation of alternative cleavage cascades (e.g., stepwise reduction of the resulting *Aβ*-peptide length as 48 > 45 > 42 vs. 49 > 46 > 43 > 40). Consequently, the *Aβ*_1–42_/*Aβ*_1–40_ ratio can increase due to the alternative cleavage pathway (starting from the C99 ε-site position ε48 rather than ε49) becoming more preferable.

Despite the extensive efforts, due to the large size and conformational mobility of the γ-secretase complex, its structure was only recently resolved with the advancement of protein engineering and cryo-EM techniques. In [99], baculoviral protein expression in insect cells was used to express and reconstitute mature and functional γ-secretase complex with the degree of glycosylation, which is involved in substrate selection, corresponding to that observed in mammal cells. The structural studies culminated in the determination of the structure of the γ-secretase complex with one of its substrates, APP TM domain, with the resolution of 2.6 Å [100]. In order to maintain the complex stability, cysteine residues were introduced into both sequences by means of Q112C mutation of PS1 and V695C mutation of APP-C83. Additionally, Asp385 catalytic residue in PS1 was mutated to Ala in order to prevent substrate cleavage. Since catalytic activity is also required for presenilin autoproteolysis, it was expressed in the form of N- and C-terminal fragments NTF and CTF. The APP TM domain in the complex closely interacts with five neighboring TM segments of presenilin (TM2, TM3, TM5, TM6, and TM7), including the segments hosting a majority of the known pathogenic mutations. The complex formation is accompanied by the unfolding of the APP helix on the C-terminal side between T719 and V721 and the assembly of an intermolecular β-sheet between V722-K725 APP residues, NTF C-terminus (residues 287–290), and CTF N-terminus (residues 377–381). Thus, the APP C-terminal segment forms a β-strand, combining with two induced presenilin β-strands into the β-sheet and ultimately presenting the peptide bond immediately preceding the β-sheet for γ-secretase catalytic cleavage.

The large number and diversity of vital processes dependent on γ-secretase activity [101] imply that any AD treatment strategy targeting the enzyme itself would most likely entail unacceptable side effects. However, knowledge of the structural and dynamic bases of γ-secretase complex interaction with *Aβ* precursors can be used to selectively influence *Aβ* production rate and/or distribution between different cleavage products.

## 5. *Aβ* Peptide Functional Activities

APP proteolysis results in the production of over a dozen different *Aβ* peptide fragments, their lengths ranging from 38 to 53 amino acid residues (based on Uniprot.org data).

*Aβ* concentrations in cerebrospinal fluid are normally within the range of 4–50 pM, the peptide remaining monomeric up to the concentrations of ~3 uM [102]. Extracellular concentrations reach 200 pM, which is still substantially below the spontaneous oligomerization threshold. The biological roles of this peptide are yet to be fully understood, but a number of studies suggest that rather than being neurotoxic in physiological concentrations, it has neuroprotective and neurotrophic neuroprotective actions in trophic deprived conditions [103,104,105]. *Aβ* may have an important physiological role in synapse elimination during brain development. Inhibition of endogenous *Aβ* production by exposure to inhibitors either of β- or γ-secretases in primary neuronal cultures caused neuronal cell death [106]. Multiple lines of evidence also link several *Aβ* species to neuroplasticity and memory formation functions. Thus, high-affinity binding between *Aβ*_1–42_ peptides and α7-nicotinic acetylcholine receptor (α7-nAChR), a Ca^2+^-permeable ion channel expressed in the hippocampal and cortical tissues, either inhibits or activates α7-nAChR signaling in a concentration-dependent manner [104]. At normal concentrations (picomolar range), *Aβ* peptides positively regulate presynaptic release at hippocampal synapses and facilitate learning by activating α7-nAChRs, whereas when the level of *Aβ* is low or high (nanomolar range), *Aβ* peptides either cause deficits in presynaptic function or abolish learning via interactions with α7-nAChRs. In [16], *Aβ* at concentrations of 0.1–1 uM (considerably exceeding the physiologically normal levels) was shown to have neuroprotective properties, suppressing NDMA-mediated excitotoxicity in neuron cultures, activating IGF-1 and insulin receptors, and causing activation of the PI-3-K signaling pathway. Prion protein PrP, normally mediating multiple trans-membrane signaling processes associated with hematopoietic stem cell replication and neuronal differentiation and performing neuroprotective functions, also has a high affinity for *Aβ* oligomers. Recent investigations indicated and suggest that this interaction between *Aβ* oligomers and PrP^C^ may impact synaptic plasticity functions and may play an important role in the pathogenesis of AD. There is a connection between AD and PrP^C^ levels, and its deficiency confers resistance to the synaptic toxicity of oligomeric *Aβ* in mice and in vitro in hippocampal slice cultures [107].

*Aβ* apparently plays multiple roles in immune function. Indeed, several new lines of evidence indicate that *Aβ* may function within the innate immune system as an antimicrobial peptide (AMP), an ancient class of peptides with potent and broad-spectrum antimicrobial activity. In vivo, the peptide was shown to perform protective functions, ameliorating the course of the diseases caused by *Salmonella typhimurium* in mice and *Candida albicans* infection in *C. elegans* worms expressing human peptide [19]. *Aβ* appears to be an essential part of normal inflammatory processes. The scavenger receptor for advanced glycation end products (RAGE), a transmembrane protein belonging to the immunoglobulin superfamily, specifically binds monomeric and oligomeric *Aβ*, causing activation of a complex downstream signaling cascade mostly associated with inflammatory processes and oxidative stress induction [108]. *Aβ* interactions are not limited to plasma membrane surface receptors, its effects of mitochondria having especially pronounced consequences. Both *Aβ* and tau proteins act synergistically to induce mitochondrial dysfunction, this effect being aggravated in aged mice in the presence of plaques and tangles [109].

Thus, *Aβ* peptides have multiple and diverse functional roles related to neuronal cell homeostasis in healthy organisms and factor into the development of several seemingly unrelated pathological processes, any of which can potentially advance to develop clinical symptoms known as Alzheimer’s disease. Such a diversity and complex interrelation of *Aβ* normal functions and roles in abnormal processes make it highly unlikely that an AD treatment strategy targeting any of its interactions individually can be effective, suggesting a focus on affecting *Aβ* production cascades and using its structure and dynamic properties to modulate its interactions with various targets and counteragents.

## 6. Structural Properties of *Aβ* Monomers and Aggregates

Although amyloid fibrillary plaques are a known hallmark of AD, they are not necessarily the main cause of neurodegeneration. *Aβ* coexists in a number of various forms, each of them potentially having both beneficial and deleterious effects depending on the current state of diverse targets and cell systems they interact with. These forms include soluble monomers of different lengths of the residual hydrophobic TM span, dimers, oligomers, protofibrils, and ultimately the fibrils depositing to form plaques.

**Figure 2:**
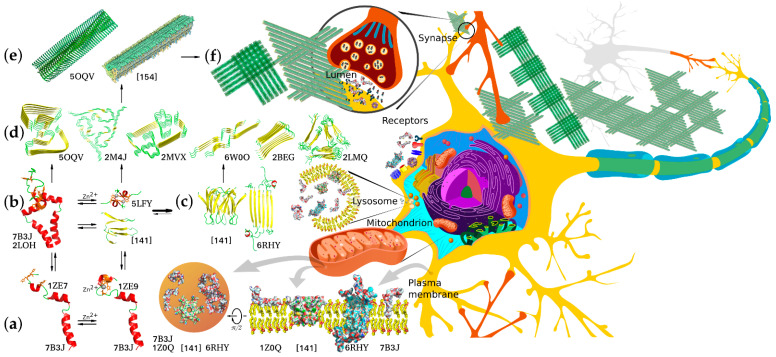
Structural diversity of *Aβ* aggregation states, aggregation pathways, and examples of biologically relevant interaction targets. Corresponding PDB accession numbers are indicated alongside the diagrams. (**a**) Free and cation-bound monomeric *Aβ* peptides (the structure combined from solution NMR structures of folded *Aβ*_1–16_ metal-binding domain and *Aβ*_17–42_ fragment) are capable of dimerizing via different dimerization interfaces situated in the TM, JM, and metal-binding site regions using protein–protein and protein–cation interactions involving α-helix, β-strand, and random coil structures. (**b**) Alternative dimers nucleate aggregation into neurotoxic intermediate oligomers that can interact with different target proteins and lipid membranes of neurons. (**c**) Two known configurations of predominantly β-structured minor oligomers capable of forming pore-like proteolipid aggregates and prone to further aggregation into protofibrillar structures. (**d**) Diverse structural motifs that can constitute amyloid fibril core structures capable of further aggregating into filaments and fibrillary deposits. (**e**) Two possible alternative fibril structures with biaxial and triaxial symmetry depositing to form macroscopic aggregates (**f**) constituting senile plaques, a known hallmark of AD. All the diverse *Aβ* aggregation forms appear to interact with multiple alternative targets, thus mediating normal physiological functions or pathological processes. The interactions are known to occur in multiple morphological units and organelles, including plasma and synaptic membranes and inter- and intracellular components.

Knowing the biophysical bases of transitions between these forms is crucial for understanding the detrimental effects of abnormal *Aβ* accumulation and finding ways to preclude or ameliorate these effects. Soluble monomeric products of APP proteolysis can adopt several alternative conformations depending on their length, hydrophobic properties, and local environment. The amino acid residues corresponding to the APP JM region can form a flexible amphiphilic helix with a propensity to submerge under the membrane surface or a β-strand, and this transition can underlie the nucleation of amyloid aggregates. The peptide dimerization and formation of higher oligomers can also occur through the metal-binding domain and be modulated by intra- and interprotein Zn^2+^ binding, or, in the case of longer *Aβ* species, through the residual TM parts within the membrane. Accordingly, the APP products of different lengths differ in dimerization modes and constants, propensity for further aggregation, and solubility of the resultant aggregates. Thus, monomers and minor oligomers can either be incorporated into the membrane, potentially causing its permeabilization [110,111], or further aggregate into larger protofibrils and mature fibrils constituting the major component of amyloid plaques. Importantly, different forms of *Aβ* can participate in aggregation, combinatorically increasing the diversity of aggregates and their properties (Figure 2).

Determination of the monomeric *Aβ* spatial structure is complicated by its oligomerization occurring at the concentrations needed for the application of NMR spectroscopy methods. This issue can be circumvented by using water/hexafluoroisopropanol and water/trifluoroethanol mixtures as solvents. At water contents below 80%, the peptide structure consists of two α-helices connected with a short loop [112], similarly to the structure of the APP TM domain [54]. An increase in water fraction results in the peptide transition to β-conformation followed by oligomerization [113].

Structural studies of dimeric *Aβ* are complicated by the same oligomerization issue, further aggravated by the fact that the dimers serve as nucleation centers for oligomerization and fibrillation [114]. Two generic types of *Aβ* are known to date: an α-helical structure similar to the conformation of TM domain in membrane-mimicking environments [115] observed in relatively unpolar solvents, such as hexafluoroisopropanol and trifluoroethanol mixtures, and a β-strand structure roughly representing a single link of a β-amyloid fibril. The spatial structure of *Aβ* dimer in solution in β-conformation obtained in [116] through 1700 ns simulation yielded an estimate of the dimerization energy at about 5 kcal/mol, which is consistent with the strong tendency of *Aβ* to aggregate in solution.

Presently, *Aβ* neurotoxicity is widely believed to be primarily associated with its oligomeric forms [117,118,119], with oligomer toxicity exceeding that of both the monomers and the mature fibrils [117]. Based on the outcomes of multiple independent studies, medium-sized oligomers produce the most significant detrimental effects, which decrease at higher molecular weights of the complex. There is no universally agreed upon predominant mechanism of *Aβ* oligomer toxicity to date, with several alternative hypotheses supported by the accumulated array of experimental evidence. The amphiphilic nature of the peptide suggests direct interactions with cellular membranes as one of the possible mechanisms, and this possibility is being widely explored. Exposure to oligomeric *Aβ* was shown to cause an increase in calcium flow across the membrane [120] inhibited by submillimolar concentrations of Zn^2+^ and Cu^2+^ ions [121]. According to some reports, the channel-like structures formed by *Aβ* oligomers [122,123,124] can influence the electrical potential of the neurons [125], and short-term memory impairments induced by *Aβ* oligomers can be caused by an increase in the local concentration of Zn^2+^ in the presynaptic region [126]. Electrophysiological measurements revealed that *Aβ*_1–42_ peptides can form at least three different types of ion channels differing in permeability, whereas *Aβ*_1–40_ peptides form no ion channels [127]. The ability of *Aβ* to form membrane pores was experimentally observed in a number of studies [123,127,128], while molecular modeling predicted the ability of *Aβ* to create transmembrane channels in interaction with cholesterol [129]. *Aβ* and its oligomers are also capable of specific interactions with certain lipid membrane components, such as cholesterol and gangliosides. As is often the case, such interactions have bilateral effects—the specific lipids in the neighborhood induce changes in *Aβ* oligomerization processes and behavior of oligomers, while *Aβ* interactions modulate local lipid membrane physicochemical properties, affecting through them functioning of membrane-associated protein machinery, both individually and within proteolipid platforms [130,131,132]. As reported in [133], trodusquemine, a naturally occurring aminosterol belonging to a family of compounds able to displace proteins from membranes, both accelerates *Aβ*_1–42_ aggregation and inhibits its toxicity, possibly through an increased rate of conversion of oligomers into less toxic mature fibrils.

Another *Aβ* feature undoubtedly associated with its neurotoxic properties is its ability to chelate transition metal ions, including Al^3+^, Fe^3+^, Zn^2+^, and Cu^2+^. The metalloproteins resulting from interaction with iron and copper catalyze redox reactions and generate reactive oxygen species (ROS), thus initiating oxidative stress, whereas zinc does not support this mechanism and even inhibits the generation of ROS [134]. It has also been shown that the addition of monomerized *Aβ* peptide even at high micromolar concentrations causes only an insignificant increase in the ROS generation in the culture of human neuroblastoma cells cultivated under physiological conditions of 5% CO_2_ (as in the body), and it attenuates radiation-induced ROS [135]. Besides that, metal cation coordination was shown to play a role in the initial stages of *Aβ* oligomerization and to facilitate its adsorption on lipid membrane surfaces through the formation of salt bridges with negatively charged lipid headgroups. The metal-binding moieties of *Aβ* are also likely to participate in the formation of channel-like structures with cation selectivity. These mechanisms appear to be responsible for neurotoxicity exhibited by *Aβ* species in comparison with P3 peptides devoid of the metal-binding segment.

Investigation of the structures and properties of *Aβ* oligomeric forms requires extensive care and efforts needed for obtaining monodisperse samples of individual oligomeric forms with sufficient stability for the application of structural methods. Several models of the formation of stable oligomers, membrane pores, and channels have been developed with the aid of molecular modeling, atomic force microscopy, electron microscopy, and electrophysiological and other methods. Among them is a model of *Aβ*_1–42_ hexamer forming a β-barrel and further assembling into a 36-membered aggregate capable of spanning the membrane and permeabilizing it for ions, including Ca^2+^ [136]. The oligomeric state of *Aβ* peptides is essentially transient, at least in vitro, the characteristic lifetimes ranging from several hours to several days [137,138]. Therefore, special experimental techniques are employed to stabilize the oligomers in the structural studies. For example, in the case of *Aβ*_17–36_ peptide, additional stabilization of the kink required for interaction of its N- and C-terminal parts with a disulfide bridge allowed resolving its dodecamer structure by X-ray diffraction method (PDB: 5HOY) [139]. *Aβ*_17–36_ dodecamer is composed of four trimeric modules and can assemble into transmembrane pores with the luminal radius of ~1 nm and the external diameter of ~11 nm. It takes five dodecamers to shape the annular pore structure. The oligomers used in this study for crystallization bind with A11 antibodies known to interact with *Aβ* oligomers, including 56 kDa dodecamers, but not with fibrillar *Aβ* [140]. This corroborates the validity of the model and obtained results.

The inherent instability of protofibrils converting into fibrils greatly complicates their structural investigations. To circumvent this problem, *Aβ*_1–42_cc peptide was engineered to be incapable of forming mature fibrils due to a disulfide bond locking it in fibril-incompatible conformation, which does not prevent protofibril formation [141]. Conformation and packing of such protofibrils were investigated by solid-state NMR spectroscopy, revealing the formation of hexameric barrel-like oligomers within the protofibril with residues 16 to 42 of *Aβ*_1–42_cc participating in intermonomeric contacts. The core of the oligomers consisted of all residues of the *Aβ* central and C-terminal hydrophobic regions, with hairpin loops extended from the core. The model accounts for why *Aβ*_1–42_ forms oligomers and protofibrils more easily than *Aβ*_1–40_. Protofibrils formed by *Aβ*_1–42_cc are indistinguishable from wild-type Ab42 protofibrils with respect to many properties: size and morphology as observed by electron microscopy and atomic force microscopy, binding of conformation-specific antibodies and the ANS dye, circular dichroism and infrared spectra, the ability to induce apoptosis in neuroblastoma cell lines, and the ability to attenuate spontaneous synaptic activity in primary neurons [141,142].

Solution NMR studies using DPC as a membrane-mimicking environment revealed an entirely different structure of the oligomeric subunit formed by *Aβ*_1–42_ (PDB: 6RHY) [143]. In these conditions, peptides formed tetramers consisting of structurally distinct subunits. The first subunit type comprises a β-hairpin made of two β-strands—β1 and β2, G9 through Ala21 and Gly29 through Val40, respectively. In the subunits of the second type, Leu17 through Phe20 residues form a short α-helix, whereas Gly29-Ile41 make up another β-strand, β3. The tetramer core thus consists of six β-strands linked with only two β-turns, along with two short and two long (including an α-helical segment) flexible N-terminal sequences. Additional experiments were performed with the use of a mixture of *Aβ*_1–42_ and *Aβ*_17–42_ devoid of the segment forming the α-helix in type two subunit and incapable of folding into the β-hairpin of the first type. With the aid of molecular modeling and SEC/IM-MS, the resultant tetramers were found to be able to spontaneously form β-sandwich octamers [143]. Molecular modeling augmented with electrophysiological measurements demonstrated the ability of these oligomers to form conductive pores in lipid bilayers. Molecular dynamics simulations [144] allowed mapping possible transitions between several predominant geometrically diverse conformation states of tetrameric *Aβ*, having substantially lower β-structure content compared to fibrillary *Aβ*.

*Aβ* aggregation into fibrillary structures depositing in the form of senile plaques has not been directly linked to any of major AD symptoms and may be even viewed as a relatively safe disposal pathway for excessively accumulated *Aβ*. The fibrillation rates are strongly dependent upon *Aβ* concentration, and *Aβ*_1–42_ can nucleate rapid aggregation of slow-aggregating solutions of other isoforms [145,146]. Extremely high mechanical and thermodynamic stability of mature fibrils in comparison with oligomers deduced from multiple simulations and obtained experimentally [147,148,149] indirectly confirms this notion. Structural information about the fibrils was first obtained by solution and solid-state NMR methods [150,151,152,153,154] augmented by electron microscopy. Several distinct structural patterns were identified with different global folds characterized by uni-, bi-, or triaxial symmetry, sharing a common β-strand–β-turn–β-strand motif, as well as stabilizing intra- and intermolecular contacts between parallel or antiparallel β-strands. In some structures, the residues constituting *Aβ* metal-binding domain were disordered; in others, they were elements of common β-structure. A similar structure was obtained by solid-state NMR when biological material from the brains of patients with clinically confirmed AD diagnosis was used as seeds for growing the fibrils [152]. This corroborates the biological relevance of the information obtained in other structural studies, taking into account the differences associated with the use of diverse *Aβ* species, initial oligomeric states, and polymerization conditions [153].

Cryo-electron microscopy (cryo-EM) (4.0 Å) structure of *Aβ*_1–42_ fibril composed of two intertwined protofilaments was recently obtained by cryo-electron microscopy (cryo-EM) complemented by solid-state nuclear magnetic resonance (NMR) experiments (PDB: 6RHY) [155]. The backbone and nearly all sidechains were clearly resolved, including the entire N-terminal metal-binding domain, which is a part of the cross-β structure, resulting in an overall “LS”-shaped topology of individual non-planar dimeric subunits of the fibrils. The dimerization interface protects the hydrophobic C-termini from the solvent. The regular helical symmetry has direct implications for the mechanism of fibril elongation and results in distinct binding sites for monomeric *Aβ*, including contacts across different subunit layers. The unique staggering of the non-planar subunits results in markedly different fibril ends, termed “groove” and “ridge”, leading to different binding pathways on the fibril ends, which has implications for fibril growth.

Thus, the development of the methods of investigation of biomolecule spatial structure allowed obtaining a detailed description of the molecular mechanisms of production of β-amyloid peptides, including the sequence of the events occurring during sequential proteolysis of APP protein. A number of factors contributing to AD development identified based on the accumulated structural information can be used to guide future development of effective compounds for the early diagnosis and effective treatment of AD.

## 7. Protein-Protein Interactions Targeting Pathological Traits of *Aβ* Forms and Aggregates

**Figure 3:**
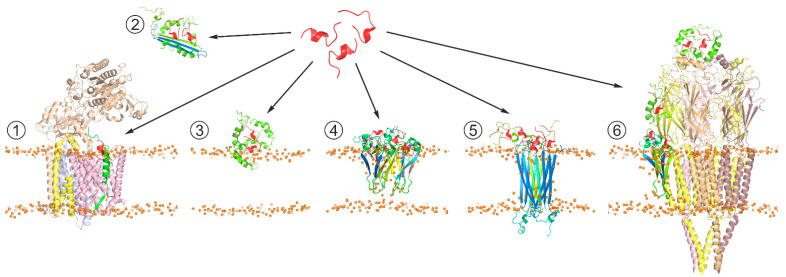
Schematic representation of the generic strategy for search of peptide-based therapeutic agents affecting *Aβ* production pathways and/or suppressing neurotoxic effects of *Aβ* oligomers. The therapeutic agent (shown in red) can (**1**) act at the stage of APP processing through interaction with *Aβ* precursors (in green) processed by γ-secretase complex (PDB: 6IYC), modifying production of mature *Aβ* isoforms; (**2**) bind soluble *Aβ* aggregates, suppressing their toxicity; (**3**) interfere with interactions of toxic *Aβ* aggregates with membrane surface, inhibiting their conversion into β-structured membrane-bound oligomers; (**4**) modify toxic properties of membrane-incorporated *Aβ* oligomers associated with membrane permeabilization [140]; (**5**) target pore-like transmembrane structures formed by *Aβ* oligomers (PDB: 6RHY), e.g., inhibiting transmembrane transport of cations; and (**6**) prevent *Aβ* from inducing abnormal functioning of soluble and membrane-associated proteins, e.g., nicotinic acetylcholine receptor (PDB: 2BG9), which can be inhibited by diverse *Aβ* oligomers in different manners.

Although *Aβ* has not been positively proved to be the main causative agent of AD, *Aβ* production or aggregation is often targeted in therapy development, and hence a lot of AD therapeutic research has been focused on *Aβ* and its life cycle [156,157,158,159,160]. Inhibition or modulation of APP secretase cleavage as AD therapy appears extremely difficult to safely achieve due to the relatively low specificity of secretases themselves and the necessity of *Aβ* production for multiple normal neuronal processes. In the light of the growing evidence of intermediate *Aβ* oligomers being the primary neurotoxic agents, the compounds able to affect *Aβ* aggregation are being increasingly intensively explored. The accumulated results of structural investigations indicate that *Aβ* aggregation is essentially driven or modulated by protein-protein and protein-lipid interactions, along with metal cation chelation. Among these, protein-protein interaction appears to be the most promising target for achieving the required selectivity, including discrimination between different *Aβ* aggregation forms and conformations (Figure 3). From a practical standpoint, short peptides possess several unique attractive properties compared to other small molecule drugs or to biological macromolecules, including a larger adoptable interaction interface with higher affinity and specificity than in small molecules, lower immunogenicity, lower manufacturing cost, ease of delivery to destination tissues, and greater access to chemical diversity than in antibodies [161,162]. In the search for peptides (or peptide-derived compounds) that inhibit *Aβ* aggregation, at least three general approaches have been taken.

In one approach, peptides are designed to interact with *Aβ* through self-complementary sequences, with the most common targets currently including *Aβ*_16–20_ KLVFF and *Aβ*_31–34_ IIGL sequences situated in the key amyloidogenic APP JM region and the N-terminal half of the APP TM segment. Numerous applications of variations of this approach have been published, intended to perturb toxic *Aβ* oligomer formation [163]. Alternatively, the search for anti-*Aβ* peptides can be based on using proteins that are known to be natural *Aβ* binders as templates, including full-length naturally occurring neuroprotectors and fragments of receptors capable of recognizing *Aβ*. Specifically, a cyclic peptide corresponding to the *Aβ* binding domain of the transthyretin, a stable homotetrameric transport protein circulating in blood and cerebrospinal fluid, has been shown to be neuroprotective against *Aβ* toxicity in vitro and suppress *Aβ* aggregation [164,165]. One more example of such decoy peptides could be represented by a peptide corresponding to the 60–76 sequence from the V-domain of the receptor for advanced glycation end products (RAGE) [166,167]. Its therapeutic efficacy is likely to be related to its competition with the RAGE V-domain for binding to *Aβ* and involvement in *Aβ* clearance in the brain. Likewise, the HAEE tetrapeptide corresponding to residues 35–38 of nAChR located in the extracellular part of α4 subunit can prevent nAChR interaction with *Aβ* by binding to the *Aβ*_11–14_ metal-binding region. Thus, it is suggested as a potential therapeutic for the treatment of α4β2 nAChR-dependent cholinergic dysfunction in AD [168]. Another strategy is based on the screening of peptide libraries for binding to full-length *Aβ*, often using phage display or mirror-image phage display techniques [163]. Library screening requires little or no prior knowledge of desired sequence or structural features. A unique advantage of the mirror phage display process is its ability to identify peptides solely consisting of d-enantiomeric amino acid residues, which makes them highly resistant to proteases, with a consequential dramatic increase in their in vivo lifetime. Additionally, d-enantiomeric peptides can be absorbed systemically after oral administration. The immunogenicity of d-enantiomeric peptides is reported to be reduced in comparison to l-enantiomeric peptides [169]. Such peptides were shown to bind the amyloid form of *Aβ* and label amyloid plaques in AD brain slices; they can also serve as carrier proteins for plaque treatment and in vivo imaging. Many of the peptides derived to date belong to a broad class of β-sheet breakers, aiming to interfere with the *Aβ* fibrilization process. An interesting alternative is illustrated by a highly specific d-enantiomeric ligand for *Aβ* identified through screening of a randomized library of over a billion dodecamer peptides, referred to as D3 [170,171,172]. This peptide, along with a number of its more recently obtained derivatives, is capable of transient protein-protein interactions with certain *Aβ* forms in an IDP/IDP (intrinsically disordered peptide) manner [173], which allows adapting to various potentially toxic oligomeric forms of *Aβ*, disfavoring formation of intermolecular contacts between *Aβ* monomers.

The recent progress in the investigation of structural properties of APP and its derivatives, their aggregated forms, and their complexes with relevant biological and pharmaceutical molecules has notably advanced our understanding of the molecular bases of pathogenic processes associated with AD and has drawn a clearer demarcation line between them and the normal functions of APP processing products. Besides suggesting new classes of potential therapeutic and diagnostic agents, this information is also crucial for identifying potential traps and pitfalls associated with undesirable interference with essential normal functions that would make the designed pharmaceuticals unsafe to use.

## Data Availability

Not applicable.
